# Family caregivers’ perceptions and challenges in the care of pressure injuries in daily life: a qualitative study

**DOI:** 10.1186/s12877-025-06114-1

**Published:** 2025-07-03

**Authors:** Yi Chen, Tingting Cai, Ping Le, Lijuan Zhang, Yunxia Chen, Linzhu Wu, Kejian Chen, Changrong Yuan, Yan Lu

**Affiliations:** 1 School of Nursing, Fudan University, No.305, Fenglin Road, Xuhu District, Shanghai, China; 2 Outpatient and Emergency Department, The First Affiliated Hospital of Soochow University, No. 188, ShiZi Street, Gusu District, Jiangsu Province, Suzhou, Jiangsu China; 3 Department of Respiratory and Critical Care Medicine, The Fourth Affiliated Hospital of Soochow University, Suzhou, Jiangsu China

**Keywords:** Pressure injury, Family caregivers, Daily life, Challenge, Field research

## Abstract

**Background:**

Pressure injuries are common in home care settings and can severely impact older adults’ quality of life. Family caregivers play a vital role in managing these injuries, yet their limited knowledge and skills may lead to delayed treatment and worsened outcomes. This qualitative study explores family caregivers’ perceptions and challenges in managing initial pressure injuries during daily caregiving. It examines discrepancies between actual caregiving practices and guideline-recommended prevention and treatment strategies.

**Methods:**

From June to December 2023, 17 older patients with pressure injuries were observed and 17 family caregivers were recruited. A qualitative design combining participant observation and in-depth interviews was employed. Field notes and interview data were analyzed using a three-level coding approach. Qualitative data analysis was performed in NVivo 14 using an inductive thematic analysis method.

**Results:**

Eight key themes emerged from the analysis. Four themes related to caregiving practices: (1) food preparation and feeding assistance, (2) hygiene maintenance, (3) repositioning and use of supportive devices, and (4) medication and dressing application. Two themes reflected caregivers’ perceptions: (1) a sense of duty and compensatory companionship, and (2) feelings of grief and helplessness. The remaining two themes highlighted major challenges: (1) identifying and responding to early signs of pressure injury, and (2) limited access to professional care and counseling resources.

**Conclusion:**

This study examined the real-world practices of family caregivers in managing initial pressure injuries, revealing both alignment with and divergence from clinical guidelines. While caregivers actively engage in core caregiving tasks, inconsistencies arise due to limited knowledge, emotional burden, and inadequate resources. A strong sense of responsibility—often accompanied by grief and helplessness—shapes their decisions and actions. Notably, caregivers frequently struggle to recognize early warning signs of deterioration, leading to missed opportunities for timely intervention. These findings highlight key discrepancies between actual care and clinical expectations. To bridge this gap, it is essential to enhance caregiver education, improve access to professional support, optimize the home care environment, and implement comprehensive, multi-level support systems.

**Supplementary Information:**

The online version contains supplementary material available at 10.1186/s12877-025-06114-1.

## Background

Pressure injury refers to local damage to the skin and/or soft tissue caused by pressure or combined shear forces. These injuries often occur in bone prominences but may also be associated with medical or other devices [1]. Depending on the setting, they are classified as community-acquired pressure injuries (CAPI) and hospital-acquired pressure injuries. CAPI include injuries that occur in nursing homes and private residences. Internationally, based on the degree of skin and tissue loss, pressure injuries are typically classified into stages 1 to 4, as well as unstageable pressure injury, and deep tissue pressure injury [1]. With the aging population and shifting disease patterns, pressure injuries remain prevalent and present an ongoing challenge for nursing care worldwide [2]. Affected individuals often experience pain, exudate, odor, and other symptoms that severely impact their quality of life. Family caregivers play a vital role in home-based care, providing essential support in rehabilitation, nutrition, emotional wellbeing, and wound management. Their involvement significantly influences wound healing outcomes and patients’ overall quality of life [3]. Although family caregivers play an important role, their lack of knowledge, care skills, early warning and identification ability, and attention to pressure injuries can result in delayed medical treatment and neglect of long-term care and home-based management, exacerbating the progression of these injuries [4].

According to interpersonal interaction theory/dualistic coping theory, family caregivers and patients support each other, and family caregivers can be sources of stress or providers of positive resources. When equipped with adequate coping and emotional regulation abilities, they are better positioned to support pressure injury recovery [5]. Family caregivers deal with the initial occurrence of pressure injuries as stressful events. In many cases, first-time family caregivers lack experience and may easily overlook critical signs, thereby hindering the recovery of pressure injuries and delaying access to timely professional care. The following questions need to be addressed: What is the current status of care provided by family caregivers for initial pressure injuries? What discrepancies exist between actual care and professional expectations? What are the warning signs that aggravate or suggest changes in conditions that are easy to miss in this type of care?

To answer these questions, this project aims to understand the real-world experiences of family caregivers in managing initial pressure injuries [6]. Additionally, we further explore and identify the discrepancies between the actual care provided to this population group and the methods of prevention and treatment of pressure injuries recommended by the guidelines, as well as the difficulties in practical nursing care for initial pressure injuries. This is of great significance in helping family caregivers to recognise, prevent, and provide cooperative treatment of pressure injuries.

## Methods

### Study design

In 2023, we used a qualitative approach using field research. In-depth semi-structured interviews with family caregivers of patients with pressure injuries and observation as complementary data collection techniques were carried out in Suzhou, China.

Observation method allowed for the collection of non-verbal and situational data that could not be fully captured through semi-structured interviews alone. To gain a deeper understanding of caregivers’ behaviors and decision-making processes, this study adopted a participant observation approach from the outset [7]. Participant observation, as used in this study, refers to the researchers’ role as both observers and minimally involved participants within the natural caregiving environment. While efforts were made to remain unobtrusive and avoid disrupting routine practices, the researchers occasionally engaged in conversations or posed questions when clarification was needed or when participants sought feedback.

To be specific, during the research process, the integration of the researchers’ focused observations into the results is mainly manifested in the following two crucial aspects. Firstly, in the data collection stage, the researchers closely monitored a series of operational details of family caregivers when caring for patients with pressure injuries, including but not limited to wound treatment techniques, the arrangement of nursing time, and the interaction methods with patients. These observed findings directly constitute an important part of the original data, providing rich materials for subsequent analysis. Secondly, within the data analysis phase, a profound and detailed analysis was carried out on the content that had been documented during the focused observations. For instance, through the classification and organization of the observed data pertaining to different family caregivers, specifically in relation to the frequency, intensity, and the types of cleaning supplies utilized in skin cleaning, the crucial behavioral elements that might be identified, which can validate the extracted themes.

### Participants and settings

A community with high proportion of older adults, compared to other communities in the city, was purposively selected as the study site. Participants were then recruited from within this community.

#### Participants

This study employed purposive sampling to recruit participants who were most suited to provide rich, relevant insights into the experience of caring for individuals with pressure injuries in community settings. The target participants were primary family caregivers, defined as individuals who provided the longest duration of care each day and were caring for someone who had developed pressure injuries for the first time. This criterion was considered the most important for inclusion, as these caregivers were best positioned to reflect on the challenges of initial care engagement, decision-making, and adaptation to caregiving responsibilities.

The inclusion criteria for participants were as follows: (1) providing care for an individual with pressure injuries for the first time; (2) the primary caregiver for the patient; (3) aged 18 years or older; and (4) informed consent to participate. Exclusion criteria included those with a diagnosed mental disorder or inability to communicate effectively.

Participants were recruited between May and October 2023 from a community setting. In this study, “initial pressure injuries” refers to the first occurrence of pressure injuries in the patient’s caregiving trajectory, regardless of stage classification.

For context, the National Pressure Injury Advisory Panel (NPIAP) defines pressure injury stages as: Stage 1– non-blanchable erythema, Stage 2– partial-thickness skin loss, Stage 3– full-thickness skin loss, Stage 4– full-thickness tissue loss, unstageable pressure injury, and deep tissue pressure injury (DTPI). These stages reflect the depth and severity of tissue damage at a given time and do not imply linear progression.

#### Participant recruitment

Potential participants were recruited through referrals from healthcare providers (YX.C., P.L., LZ. W., and LJ. Z.), who had access to local community service centers. Specifically, these patients were discharged from a tertiary-level hospital and returned home, where they typically did not receive routine follow-up care from general practitioners. When patients with pressure injuries meeting the study criteria were identified, these providers promptly informed the researcher (Y.C.) and facilitated initial contact with their family caregivers. The researcher then explained the study to interested individuals, providing detailed information about its purpose, procedures, and their rights as participants.

Participant selection was carried out by the research team, based on caregivers’ active involvement in pressure injury care. From the pool of eligible candidates, the research team purposively selected participants to ensure a diverse range of caregiving experiences, prioritizing those capable of offering rich, in-depth perspectives on the perceptions and challenges involved in daily care. Informed consent was obtained from both the patients and their family caregivers prior to data collection.

#### Settings

This study was conducted in Suzhou, Jiangsu Province, China, situated in the central region of the Yangtze River Delta. The area has a subtropical humid monsoon marine climate. Suzhou is among the most economically developed prefecture-level cities in China. In 2024, its gross regional product reached 2,672.7 billion yuan, with an annual growth of 121.3 billion yuan, ranking first in Jiangsu Province and sixth nationwide. That year, the per capita disposable income of permanent residents was 77,524 yuan. The city consists of 46 subdistricts, with the study community comprising 8 subdistricts, 169 neighborhood committees, and 938 village committees, covering a total land area of 83.42 square kilometers. As of 2023, the resident population of this district was approximately 926,100.

China’s healthcare service system comprises three main components: medical service institutions, preventive healthcare institutions, and primary healthcare service institutions. Of these, primary healthcare institutions form the foundation of the system. These include township health centers, community health service centers, and village clinics. Embedded within local communities, they provide essential services such as basic medical care, disease prevention, health promotion, rehabilitation, and chronic disease management. These institutions are critical in ensuring access to healthcare, especially in rural and underserved areas.

Primary healthcare institutions are responsible for delivering primary preventive care, managing common and frequently occurring conditions, coordinating timely referrals for complex or severe cases, and supporting pre- and post-hospital care in collaboration with higher-level hospitals. In Suzhou’s Gusu District, there are 29 community health service institutions. The term “community nurses” typically refers to nurses working within these organizations. A key mandate of community health service institutions is to deliver integrated, community-based care that is family-centered, health-focused, and spans the full life course. These services prioritize vulnerable populations such as older adults, women, children, and patients with chronic illnesses, integrating prevention, treatment, rehabilitation, health education, and family planning guidance.

Our study site was the community located in Gusu District in the center of Suzhou, a famous historical and cultural city, and was equipped with home care service workstations and community health service institutions. However, it remains unclear how pressure injury prevention and management are being implemented within these community-based institutions, particularly in home care contexts.

#### Observation settings and aspects

Observation took place at the participants’ homes in the specific community. Observations of family caregivers primarily focused on the following aspects: (1) family caregivers’ daily activities in natural settings; we observed the specific tasks performed by family caregivers during the caregiving process, such as feeding, cleaning, and assisting patients with movement. In addition, we recorded their daily routines, including the balance between caregiving, household chores, and rest. (2) interactions among family members, we paid attention to the communication styles between the patients and their family caregivers, including verbal exchanges and emotional support. At the same time, we observed how family members collaborated to share caregiving responsibilities or handle conflicts. (3) body movements, we observed whether family caregivers adopted effective body movements during caregiving tasks, such as lifting or moving patients or using assistive devices. We also observed whether the movements were safe and whether they could lead to physical strain on the caregiver. (4) facial expressions, we analyzed family caregivers’ facial expressions to understand their emotional states, such as fatigue, anxiety, happiness, or satisfaction.

#### Interview settings and timing

The interviews were conducted in the family caregivers’ homes to help them feel more natural and comfortable. The interviewers were prepared to adjust timing based on the family caregivers’ needs or unexpected changes. We arranged the interviews at times that were convenient for the participants, considering their daily routines, or caregiving responsibilities. What’s more, we tried to avoid scheduling interviews when participants might be tired, such as late at night or after a long workday, to ensure thoughtful and engaged responses.

### Data collection and methodology

Semi-structured interviews were conducted with each family caregiver at a convenient time, typically in private and quiet settings to minimize external disturbances. Each caregiver participated in one interview session, lasting approximately 30–45 min depending on the depth of discussion. Interview questions were carefully designed to address the study’s core objectives (Supplementary file 1 Interview guide).

Observation approach consisted of three progressive stages: descriptive, focused, and selective observations. Scripts were utilized during the observation process to ensure the systematic and consistent collection of data. The scripts contained “how to prevent pressure injuries, patterns of interaction between family caregivers and patients, key aspects of workflow in the daily life, notes of other key points observed.” (Supplementary file 2.) Upon entering the field, researchers conducted descriptive observations to establish rapport - such as engaging in informal conversations with patients and family members - and to document the general context and caregiving workflow. Focused observations were conducted following preliminary communication with family caregivers and were scheduled between 07:30 and 18:30 to align with caregivers’ daily routines. Each interviewee was observed for 2–3 h in their home setting for once, covering key periods of morning and evening caregiving activities. During the observation periods, researchers primarily engaged in non-intrusive observation, taking detailed field notes on caregiving behaviors, and interactions between caregivers and care recipients. Occasional clarifying questions were asked to understand caregivers’ caregiving processes, but no direct interference in their activities occurred. Selective observation focused specifically on content related to pressure injury care. The study focused on prevention and treatment recommendations for ‘populations in the community, the elderly care, and rehabilitation settings’ in the Chinese version of the Prevention and Treatment of Pressure Ulcers/Injuries Guidelines, originally developed by the European Pressure Ulcer Advisory Panel in 2019 [1], including how to prevent pressure injuries (e.g. supports, position changes, use of dressings), nutritional support (e.g. how to provide food), how to assist professional nursing staff to treat pressure injuries. Information about care activities that occurred outside observation periods was gained through informal conversations with nurses, physicians, and patients or their caregivers. Data were recorded through shorthand field notes, audio recordings (with participant consent), and photographs.

To minimize bias associated with having multiple researchers involved in data collection, all team members received standardized training on observation and interview procedures prior to the study. A subset of data was independently coded by two researchers to assess consistency. Regular team meetings were held throughout the data collection period to discuss field experiences, address emerging biases, and ensure methodological alignment.

Although no formal post-training evaluation was conducted, frequent team discussions allowed for real-time methodological adjustments. Given that field observations cannot be retrospectively verified, researchers were encouraged to write detailed field notes and reflective memos immediately after each session to enhance data integrity. Participants were informed in advance of the researchers’ visits but were not made aware of the specific research objectives. This strategy was employed to reduce response bias and encourage natural, authentic behaviors.

The researchers who participated in the observations and interviews were professionals in the field of nursing Among them was LZ.W., possessing 10 years of experience in wound care nursing; LJ.Z., with 5 years of experience in wound care nursing; P.L., having 3 years of experience in nursing management and 10 years of experience in clinical nursing; and YX.C., who had 7 years of clinical nursing experience and had also studied relevant sociological theories. All of these individuals had frontline nursing experience and comprehensive backgrounds in qualitative research.

Data saturation was monitored throughout data collection. Recruitment was stopped when no new themes or significant variations emerged after several additional interviews. The entire data collection period spanned six months.

### Data analysis

#### Data Preparation and field reflection

Within 24 h after the end of field observation, the researchers combined the collected data and their own memories to compile field notes and recorded their reflections and perceptions of the field research for that day. Through repeated reading of the field notes, the researchers extracted problems from the original materials and reflected on them. In the subsequent field research, repeated observations, continuous thinking, and comparisons were conducted for the problems found.

#### Transcription

The audio recordings of semi-structured interviews were transcribed verbatim by designated members of the research team. To ensure transcription accuracy, each transcript was reviewed by another researcher to minimize potential errors. The transcribed textual data were imported into NVivo.14 for coding and analysis. During the data entry process, standardized naming conventions and file storage protocols were established by the research team to ensure traceability and consistency.

#### Coding and analysis

We conducted an inductive thematic analysis combining the six-phase framework [8]. The coding procedures in this study including open coding, axial coding, and selective coding [9].

First, the audio-recorded interviews were transcribed verbatim. Two researchers (Y.C. and TT.C.) independently read through the transcripts and field notes to gain familiarity with the data. During open coding, they identified initial codes from the raw text while preserving the original expressions of participants and field observations as much as possible. Open coding was performed by two researchers who repeatedly read the field notes, and the coding process presented the original content of the field notes as much as possible. A third researcher then summarized the coding results of the same field notes, documented any disputed codes, facilitated collective discussions to resolve disagreements, and, when necessary, sought expert opinions for final determination. For axial coding, we examined how caregivers’ emotions and caregiving behaviors interacted to shape their overall caregiving experiences and other researchers in the team proposed modification suggestions. To reach consensus and clarify relevant categories, selective coding involved the collaboration of two researchers to identify and establish relationships between categories, ultimately forming core category that encompassed all others.

Following the development of preliminary themes, the entire dataset was re-examined to refine and confirm the thematic structure. Subthemes were identified where appropriate, and the consistency of the themes was reviewed through team discussions and expert consultations. Coding results and interpretations were also shared with stakeholders and key informants for validation, enhancing the credibility of the findings. Finally, clearly defined themes were named to capture their core meaning, and the findings were synthesized and reported in the final manuscript.

### Data management

Data management strictly adhered to the research protocol approved by the ethics committee. All data were securely stored on encrypted hard drives with password-protected access, which was restricted to core members of the research team to maximize data security.

To validate data integrity, the following measures were adopted: firstly, regular team meetings were conducted during the data analysis phase to review key findings and coding results, ensuring scientific rigor. Secondly, selected participants were invited to confirm the accuracy of recorded data and the validity of research conclusions.

### Ethics and quality control

Before conducting the field study, the researcher provided all participants with a clear explanation of the study’s purpose. Participants were informed that their verbal expressions and caregiving behaviors in the context of daily life would be observed and used solely for academic analysis. They were assured that their identities would remain anonymous, and no real names would appear in any records or publications. Written informed consent was obtained from each participant prior to data collection. The study protocol, including this consent process, was reviewed and approved by the Ethics Committee of Soochow University (Approval No. SUDA20231020H02). We used the COREQ checklist [10] to guide reporting of the qualitative methodology and results.

To minimize the observer effect and preserve the authenticity of natural behaviors, we did not disclose the full detail of the specific research objectives (e.g. our focus on the discrepancies between the actual care provided to the patients and the methods of prevention and treatment of pressure injuries recommended by the guidelines). This limited disclosure approach was adopted in line with accepted qualitative research practices, where withholding certain research details is ethically permissible if it poses no risk, coercion, or deception.

During field observation, researchers maintained a non-intrusive stance and did not evaluate or interfere with participants’ speeches or behaviors. Observed family caregivers continued with their usual caregiving routines and participated in interviews guided by a semi-structured interview guide (see Supplementary File 1). Data were collected through detailed field notes, with occasional clarifying questions asked solely to better understand caregiving behaviors and decisions.

To enhance the rigor and trustworthiness of the study, the research team held regular seminars throughout the data collection period to reflect on field experiences, discuss emerging issues, and identify areas requiring further investigation.

## Results

### Characteristics of the field, family caregivers, and patients

A total of 17 family caregivers, aged 48–79 (57.41 ± 9.21) years, were interviewed and observed in this study. Most family caregivers had received at least six years of basic education, eight (47.06%) had been educated in primary school or lower level, five (29.41%) in junior high school, and four (23.53%) in senior high school. Fourteen family caregivers were female, including eight daughters (47.06%), three wives (17.65%), two sons (11.76%), one husband (5.88%), and three daughters-in-law (17.65%). The duration of care ranged from 2 to 204 months (Table 1). Most caregivers were from low- to middle-income households and were not professionally trained. Some caregivers may consult community nurses for informal advice, but systematic professional support for pressure injury prevention and management at home is limited.

**Table 1:** Demographic data of family caregivers

Number	Gender	Age (years)	Education background	Relationship with patient	Duration of care (months)
CG1	female	48	junior high	daughter	12
CG2	male	79	none	husband	2
CG3	female	50	junior high	daughter	6
CG4	female	45	senior high	daughter	16
CG5	female	52	junior high	daughter-in-law	3
CG6	female	47	primary school	daughter	28
CG7	female	50	junior high	daughter	6
CG8	female	56	primary school	daughter-in-law	12
CG9	female	57	none	daughter-in-law	18
CG10	male	54	primary school	son	12
CG11	female	55	primary school	daughter	15
CG12	female	71	primary school	wife	204
CG13	female	65	junior high	wife	48
CG14	female	68	none	wife	24
CG15	male	60	senior high	son	12
CG16	female	57	senior high	daughter	48
CG17	female	62	senior high	daughter	24

Abbreviation: CG = caregiver

The demographic data of patients with pressure injuries and their characteristics are shown in Table 2. The patients’ ages ranged from 68 to 86 (78.47 ± 5.35) years, including 9 female patients. The wound duration ranged from 0.5 to 12 (2.42 ± 2.71) months. The pressure injury stages were as follows: stage 4 (30.43%, 7/23), stage 3 (21.74%, 5/23), stage 2 (39.13%, 9/23), and stage 1 (8.70%, 2/23).

**Table 2:** Demographic data of patients with pressure injury and characteristics of pressure injury

Number	Gender	Age (years)	Wound duration (months)	Wound size [length (cm)*width (cm)*depth (cm)]	Stage*	Location	Total number of pressure injuries
P1	female	75	2	6*7*3	4	sacrococcygeal region	3
P2	female	78	1	7*8*4	4	sacrococcygeal region	1
P3	male	76	2	7.5*8,5*2.5	4	sacrococcygeal region	1
P4	female	78	0.5	4*4*1	2	right ankle	1
P5	female	83	3	4*5*2	4	right heel	1
P6	female	74	4	3*2*2	3	sacrum	1
P7	female	78	1	2*3*1	2	sacrococcygeal region	1
P8	female	84	4	1*2*0.3	2	right back	1
P9	female	82	2.6	2*2*1,7*7*2, 4*4*0.8, 3*4*1	2 (3), 1 (2)^#^	sacrococcygeal region, right forearm, heels, back	5
P10	male	86	1	1*1*0.3	2	left shoulder	1
P11	male	79	1	3*4*0.1, 1*1*0.2	2	sacrococcygeal region	2
P12	male	73	2	2*2*0.3	2	left hip	1
P13	male	68	12	2*2*1, 2*4*1	3,4	sacrococcygeal region	5
P14	male	70	0.5	7*10*0.8	3	sacrococcygeal region	3
P15	male	85	0.5	3*5*1, 1*2*1	3,4	back	2
P16	female	85	2	1.5*2.7*1	3	back	1
P17	male	80	2	2.8*3.5*0.8	4	left foot	1

Abbreviations: P = patient

*: stage 1; stage 2; stage 3; stage 4

#2 (3): There are three pressure injuries diagnosed as stage 2

1 (2): There are two pressure injuries diagnosed as stage 1

### Empirical findings and interpretive insights, and recommendations derived from the data and contextual Understanding

Eight themes were extracted after analysing the on-site observation records and interviews. These include four topics on caregiving practices: (1) food preparation and feeding assistance, (2) hygiene maintenance, (3) repositioning and use of supportive devices, and (4) medication and dressing application; two perception topics (1) a sense of duty and compensatory companionship, and (2) feelings of grief and helplessness, and two challenge topics: (1) identifying and responding to early signs of pressure injury and (2) limited access to professional care and counseling resources.

### Discrepancies between family and professional caregivers in pressure injury care practice

In this study, family caregivers often lacked professional knowledge of wound care and therefore relied heavily on the assessment and guidance of healthcare professionals—such as doctors and nurses—when managing the pressure injury occurring for the first time in their loved ones. For example, CG9: *“If there is no time to go to the hospital*,* I will take photos of her wounds to consult the doctor/nurses…*”.

They expected clear instructions on issues such as whether repositioning was needed, how frequently it should be done, how to clean the wound, and which dressings to use. We categorized this as an information dependency relationship. It can be reflected in the sub-themes below.

#### Sub-theme 1: food Preparation and feeding assistance

Most family caregivers demonstrated proficiency and dedication in preparing meals tailored to the patients’ dietary needs. For those who were unavailable due to work or other responsibilities, external caregivers such as nannies were arranged to ensure the provision of nutritious meals. The importance of meal preparation was emphasized not only in terms of maintaining nutrition but also as an act of care and emotional support.

Several caregivers adapted the patient’s diet based on functional limitations, such as difficulties in chewing or swallowing. For instance, soft-textured and protein-rich foods were frequently mentioned.

*“She*,* since last year*,* cannot chew*,* and so our family members will buy some protein powder and brew for her*,* basically*,* some vegetable juice*,* juice*,* rice paste*,* and sometimes along with some chicken breast puree*,* minced meat mixed together as her meals*,*”* said one caregiver (CG8), illustrating the family’s efforts to maintain nutritional intake despite functional decline.

Others outsourced meal preparation to domestic helpers to reduce the burden on elderly caregivers with chronic illnesses.

*“We ask our nanny to prepare three meals a day*,* and I won’t let my mom cook or do housework since her diagnosis of chronic obstructive pulmonary disease.*” explained another participant (CG 16).

These findings indicate that although most family caregivers are generally competent in performing food-related caregiving tasks, the specific approaches they adopt are influenced by their time availability, and the caregivers’ own daily routines, with the dietary choices tailored to the patient’s functional abilities.

#### Sub-theme 2: hygiene maintenance

While most family caregivers endeavored to uphold basic hygiene standards, nearly half (47.06%, 8 out of 17) did not follow recommended practices related to skin care and the use of sanitary materials. Observational data revealed several suboptimal behaviors, including the use of coarse toilet paper to minimize costs, application of alkaline soap instead of pH-balanced cleansers, and the use of diapers for patients who remained continent and capable of independent toileting—primarily to reduce caregiving workload.

Such practices were often rooted in long-standing habits and limited understanding of proper hygiene protocols. Caregivers showed reluctance to change familiar routines, particularly when the rationale for new practices was not clearly communicated. For instance, one caregiver (CG11) questioned the need to switch hygiene products, stating, *“We’ve been using this kind of toilet paper for years*,* and none of us ever had any problems with it. Why should we switch to wet wipes?*” This resistance underscores the importance of caregiver education that not only conveys best practices but also addresses underlying beliefs, knowledge gaps, and emotional attachments to habitual methods.

In some cases, time-saving strategies led to inappropriate care decisions. For example, one caregiver (CG8) regularly used diapers for the patient (P8) who was not incontinent but required assistance to get out of bed. This approach allowed the caregiver to complete other household duties such as shopping and administering medications. However, a lack of awareness regarding the need for frequent diaper checks led to delayed changes and subsequent skin damage, including marked redness on the patient’s hips.

These findings highlight a critical gap in caregiver training regarding hygiene maintenance and skin protection. Practices that may appear practical or efficient in the home context can inadvertently increase the risk of pressure injuries and would not meet professional care standards.

#### Sub-theme 3: repositioning and use of supportive devices

Family caregivers frequently reported uncertainty regarding how to properly reposition patients and select suitable pressure-relieving devices such as mattresses and cushions. While most caregivers were aware of the need to reposition patients to prevent pressure injuries, they lacked essential knowledge about the recommended frequency, repositioning angles, and correct application of supportive surfaces.

This knowledge gap was especially apparent in the care of patients confined to wheelchairs. Prolonged sitting without effective offloading techniques often resulted in sustained pressure on high-risk anatomical areas, including the scapulae, ischial tuberosities, sacrum, and thighs. A prevailing misconception among caregivers was that wheelchair users were more mobile and therefore less vulnerable than bedridden individuals. This bias contributed to underestimation of risk and inconsistent implementation of pressure-relieving strategies. Moreover, timely repositioning was further hindered by caregiver fatigue, multitasking demands, and lack of access to appropriate supportive equipment.

Several caregivers expressed confusion about how to select and use pressure-relieving devices. One caregiver acknowledged relying on intuition rather than a fixed schedule:

“*When P4 is sitting in the wheelchair and sunbathing*,* I just cover her knees with a quilt and then go handle other things… I usually go by my feeling or ask her if she wants to change position*” (CG4).

Caregivers commonly adopted improvised or ad hoc solutions in the absence of professional guidance. Some resorted to makeshift methods such as using pillows or self-made cushions, while others expressed uncertainty about sourcing appropriate support equipment. One caregiver stated, “*I’ve used pillows and even handmade cushions filled with foam to make her more comfortable*” (CG9). Another admitted, “*Honestly*,* I don’t even know where to find a professional*,* suitable mattress*” (CG7).

In response to worsening pressure injuries, one caregiver turned to online suggestions: “*Someone suggested an air mattress*,* so I quickly ordered one online. But is there a better solution for pressure relief?*” (CG16). This statement reflects a reactive rather than preventive approach to care and highlights the limited awareness surrounding effective pressure-relieving strategies.

To sum up, these responses reveal a significant gap in caregiver knowledge and access to reliable information regarding the selection and proper use of pressure-relieving devices. The findings underscore the urgent need for accessible, context-specific educational resources and support systems to enhance caregiver competence in pressure injury prevention within community and home settings.

#### Sub-theme 4 medication and dressing application

Family caregivers had access to a variety of topical treatments and dressings for pressure injury management; however, many lacked adequate knowledge regarding their appropriate use. In particular, decompression dressings—which are essential for effective pressure redistribution—were frequently misunderstood or overlooked. Most caregivers sourced treatments through pharmacies, social networks, or personal experience, relying heavily on familiar ointments, powders, or basic dressings.

One caregiver shared her frustration after relying on anecdotal advice: “*We heard that a special powder was effective*,* so whenever there was a small break on his buttocks*,* I would sprinkle it on. It worked at first*,* but now the wound is getting worse. I can’t control it anymore*…” (CG17).

Another caregiver drew confidence from her previous informal caregiving experience, stating, “*I’ve been a live-in nanny for many years and cared for two older adults with pressure injuries… I used a type of golden ointment that worked well*,* so when I cared for P10*,* I was confident and even shared my methods with the family*” *(The caregiver expressed visible pride.)* (CG10).

However, others expressed confusion and frustration due to inconsistent medical guidance: *“Different hospitals gave us different medications and dressings… we bought many kinds of products*,* but I didn’t see any improvement*” (CG1).

Misconceptions about wound care were common among participants. A number of caregivers believed that wounds should remain dry and exposed to air, which led to hesitation in using occlusive or decompression dressings. Financial concerns, perceived inconvenience due to frequent dressing changes, and fear of moisture retention further contributed to their avoidance.

These findings point to a persistent gap between caregiver practices and evidence-based pressure injury management. They highlight the urgent need for healthcare professionals to provide consistent, comprehensible guidance on dressing selection—particularly regarding decompression products—and to improve the availability and affordability of appropriate wound care supplies in home-based settings.

### Psychological perception of family caregivers participating in pressure injury nursing practice

The psychological processes of family caregivers involved in pressure injury nursing care are complex and contradictory. The target population who needs family caregivers to participate in the nursing practice of preventing and treating pressure injuries is often disabled, and disabled people need not only the help of family caregivers in physical care and daily life care but also more love and companionship from family caregivers.

#### Sub-theme 1: a sense of duty and compensatory companionship

Family caregivers often expressed a strong sense of obligation, shaped by traditional filial values and emotional bonds with the care recipient. For many, caregiving was not only a practical necessity but also a moral responsibility and a way to compensate for past absence or repay a lifelong debt of gratitude. This sense of duty was particularly evident in families with close intergenerational relationships, where caregivers willingly invested considerable time, emotional energy, and financial resources in care provision.

Several caregivers described caregiving as a form of redemption or delayed filial piety. One caregiver explained, *“My brother is a businessman; my mother used to take care of his family. I didn’t have time to care for my parents before*,* but now that I’m retired*,* I’ve taken them in and do my best to care for them. This is my way of showing filial piety”* (CG16).

In families with multiple adult children, caregiving was often nominally shared, but one individual typically assumed the primary role, especially for tasks perceived as complex or emotionally challenging, such as wound care. For example, CG1 stated, *“According to the doctor’s guidance*,* the dressings should be changed regularly. Our family members were supposed to take turns… but in reality*,* I am the primary caregiver… the others feel too anxious or unsure to handle the wound.”* This quote reflects a common dynamic in home-based care where traditional expectations of shared responsibility do not translate into equal task distribution.

In some cases, emotional commitment drove caregivers to provide intensive and meticulous care. One particularly dedicated caregiver insisted on staying with her grandmother around the clock and closely followed medical advice, including repositioning the patient every two hours. Others described efforts to learn about the patient’s condition, coordinate hospital visits, and maintain presence during hospitalizations as part of their perceived familial duty.

These findings underscore that for many caregivers, participation in pressure injury care is deeply rooted in cultural norms of reciprocity and filial responsibility. Caregiving becomes both an emotional expression of attachment and a compensatory act aimed at restoring balance in intergenerational relationships.

#### Sub-theme 2: feelings of grief and helplessness

In the context of pressure injury care, many family caregivers reported experiencing complex emotional responses, including sadness, anxiety, guilt, and helplessness. These emotions often stemmed not only from the practical difficulties of wound management but also from the psychological burden of witnessing their loved one’s decline.

Caregivers caring for end-of-life patients frequently described overwhelming feelings of loss and emotional fatigue. As one caregiver explained, “*My dad has been bedridden for a long time… with his life seemingly nearing its end*,* I feel overwhelmed. Fortunately*,* I have enough time these days*,* and I will do my best to care for him—after all*,* I only have one dad.*” (CG15) This statement reflects the emotional tension between anticipatory grief and a determination to provide compassionate care in the final stages of life.

Feelings of helplessness were particularly evident when early signs of pressure injuries were missed or misinterpreted, and when caregivers lacked the guidance needed to respond effectively. One caregiver recounted, “*At first*,* I thought it was just a scratch… I was in a hurry*,* without anyone to guide me. I felt very powerless and helpless.*” (CG3).

Guilt and self-blame often co-occur with, or emerge secondary to, feelings of grief and helplessness, particularly among those who had assumed a leading caregiving role. In one case, a caregiver (CG2) demonstrated emotional strain while dressing a wound, blaming himself for not preventing the injury. His silent pauses, gentle gestures, and attempts to comfort the patient during dressing changes reflected deep emotional distress, as well as a sense of personal responsibility.

These narratives reveal that the emotional toll of caregiving extends beyond physical labor to include unresolved grief, moral pressure, and psychological exhaustion. The findings underscore the importance of integrating emotional support and psychological counseling into community-based care programs for family caregivers.

### Exploring the difficulties of family caregivers in pressure injury care practice

The family caregivers are not professional caregivers. On-site observations of the 17 family caregivers and patients revealed that they faced two major challenges in the practice of pressure injury care: identifying and responding to early signs of pressure injury and limited access to professional care and counseling resources.

#### Sub-theme 1: identifying and responding to early signs of pressure injury

Seventeen family caregivers repeatedly noted the appearance of red spots or erythema on patients’ skin but did not initially recognize the significance of these signs, allowing them to progress into pressure injuries. Some caregivers even mistook the condition for eczema, assuming it could be alleviated by simply drying the affected area. For example, CG9 stated: *“I’m really good at recognizing pressure injuries*,* and I always remind other family members when I see redness in this area. I tell them to sprinkle some baby powder to keep it dry so it doesn’t get worse.”*

However, CG9’s overconfidence led to ineffective measures that did not align with clinical guidelines, and the warning signs she mentioned were not comprehensive.

CG13 believed that her husband’s skin was simply sensitive and prone to redness due to an allergy, which led her to overlook early signs of pressure injuries. Meanwhile, CG7 expressed regret for not recognizing the condition earlier: *“I was too busy a few days ago*,* and I didn’t visit as often. Then one day*,* when I went to turn her over*,* I suddenly found the wound. If I had noticed it earlier*,* it would have been easier to treat and heal.”*

These examples underscore the difficulties family caregivers face in recognizing the early signs of pressure injuries and highlight the need for better education and awareness to improve timely interventions.

#### Sub-theme 2: limited access to professional care and counseling resources

More than half of the caregivers reported a lack of timely access to professional knowledge and resources since the onset of the pressure injury. This limitation was especially pronounced among patients with comorbidities or restricted mobility, where frequent hospital visits were not feasible. These findings highlight the urgent need to strengthen community-based educational resources and expand the availability of professional care and consultation services in home settings.

One caregiver (CG16) caring for a bedridden parent with Parkinson’s disease noted the physical and logistical difficulties of traveling to medical facilities and expressed a strong need for accessible, community-based professional guidance. She emphasized that having a reliable contact at a nearby health center would greatly improve care efficiency and response time.

*“Ideally*,* there would be a professional caregiver at the community health service center near my house*,* so I could quickly ask questions about pressure injuries and save valuable time*,* ultimately improving my caregiving efficiency.”* (CG16).

These experiences underscore a broader systemic issue: the inadequacy of localized professional resources to support home-based pressure injury care. Expanding community health services and providing caregivers with direct channels to professional advice are essential steps toward improving care quality and outcomes.

## Discussion

### Inappropriate care practices by family caregivers

*When family caregivers participate in pressure injury care practice*,* they still perform inappropriate caring procedures (e.g. frequency of postural change and keeping the wound clean and hygienic)*.

According to an Indonesian study [11], despite living with family members, most CAPI patients did not receive care from them before hospitalization. While another study suggested that active participation of family caregivers in pressure injury management may improve recovery outcomes [12], this casual relationship has not been validated by longitudinal research. Mastering the caregiving skills associated with pressure injuries is mostly the responsibility of the family caregiver. Lack of attention to the role of family caregivers can lead to poor knowledge and ability of family caregivers to prevent pressure injuries, which in turn may increase the chances of patients developing pressure injuries. Rafiei et al. [5] advocated that family caregivers should be involved in the care of patients with pressure injuries, and strategies for improving the skills of family caregivers should be investigated further. In the field investigation of family caregivers in this study, most family caregivers could complete basic daily care content, especially diet, which is probably in accordance with the Chinese culture of ‘food is people’s foremost/primary concern’ [13]. It seems that in daily life, family caregivers are mainly responsible for providing food. The food they provide to patients is sufficient, nutritious, and diverse. However, the study found that the shortcomings of family caregivers’ care lie in the frequency of changes in body position and the lack of methods to maintain cleanliness and hygiene. In the eyes of family caregivers, patients are still considered normal people and are not treated as vulnerable people with damaged skin barriers. Ways to reduce pressure and clean the skin of people with disabilities are often overlooked.

### Strategy 1: Education for family caregivers of disabled older adults should include the identification of pressure injuries and related early warning signs

A retrospective study in the United Kingdom reported that 81% (*n* = 1,026) of older patients presenting with CAPIs at hospital admission were living at home, with a mean age of 80 years [14]. Once pressure injuries occur, the wound healing cycle becomes too long to predict the prognosis. This study also found that most family caregivers may miss early warning signs, potentially delaying intervention and increasing the likelihood of systemic infection; if the pressure injury is not so severe as to reach stages 3 and 4, its treatment duration and economic expenditure can be reduced. This underscores the importance of prevention. As widely acknowledged, prevention is better than cure, To achieve effective prevention of pressure injuries, educational programs for family caregivers of disabled older adults should focus on equipping them with practical skills in early detection, proper repositioning, and recognition of warning signs. In addition, in home settings, the primary responsibility for patient care typically rests with family caregivers [15]– [16]. However, the prevention and management of pressure injuries is a complex task that demands specialized knowledge and skills [17]. Evidence from Sharma et al. [18] indicates that family caregivers often lack sufficient knowledge of pressure injuries, and their care practices may be suboptimal. Empowering family caregivers in the prevention of pressure injuries can substantially reduce patients’ PI incidence [5]. Karimi et al. [19] reported that providing education and support to family caregivers following hospital discharge led to a 50% reduction in the prevalence of pressure injuries. Thus, Family caregiver play a vital role in prevention and management of pressure injury for the disabled older adults. As most family caregivers are middle-aged or elderly in an ageing Chinese society, educational materials should be tailored to their needs by using clear language and relatable case examples to increase comprehension and interest. The recommended educational content should be primarily directed toward family caregivers of patients at high risk of pressure injuries, particularly during the hospital discharge process or within the early stages of home care. To ensure accessibility, we suggest a combination of delivery methods, including brief in-hospital training sessions led by nurses before discharge, textual or visual educational materials provided to caregivers, follow-up visits or calls from community nurses to reinforce key concepts, use of digital tools (e.g., videos, mobile apps) tailored for caregivers with varying levels of health literacy [20].

### Strategy 2: To promote the availability of professional care and counselling resources for pressure injuries, the community service centers can play an important role in conducting science popularisation using various effective media

As society enters an era of advanced ageing, pressure injuries, a common complication, have attracted the attention of researchers. Economic considerations, limited access to professional resources, and out-of-pocket medical expenses may have influenced caregivers’ decisions and contributed to the challenges they reported. To promote the availability of professional care and counselling resources for pressure injuries, the public community health service centers can play an important role in conducting science popularization using various effective media.

In China, where families have traditionally played a central role in caring for older adults and individuals with chronic conditions, the following question arises: how can communities be mobilized to support long-term home care for disabled family members? One essential strategy is to utilize effective media channels to deliver science popularization activities that raise awareness of available professional care services [21], improve caregivers’ knowledge of when and how to seek help, and ultimately facilitate timely access to professional counseling and pressure injury management resources.

Social media, including websites, forums, WeChat, and Weibo in China, have been applied largely in the field of health promotion and have become the preferred method because of their wide audience and effectiveness in promoting the dissemination of organisational and personal health information [22]. According to multiple guidelines, family caregivers should be trained on the risk factors for pressure injuries, nutrition, skin assessment, characteristics of pressure injuries, interventions to prevent pressure injuries from occurring, signs and symptoms of complications of pressure injuries (such as infections), and use of protective equipment and devices. Although family caregivers are satisfied with Internet-based guidance and report the benefit of the guidance provided, many platforms and apps are only used sporadically, and their use has decreased over time [23], which may be due to changes in the tasks assigned to family caregivers. Gaps exist between providers’ information and the needs of family caregivers. The persistently high incidence of pressure injuries and the declining use of various management strategies suggest the need to develop professional care and counselling resources for family caregivers.

In many countries, community nurses play a critical role in identifying the challenges faced by informal caregivers and patients in managing pressure injuries, as well as in delivering targeted training to address these issues. Their specialized knowledge enables them to understand the distinct difficulties encountered in various home care environments, making their contributions essential to effective pressure injury management [24], [25].

In China, community health service centers are designed to maintain close connections with local residents and serve as key providers of essential healthcare services. As core members of these centers, community nurses are expected to be central to the prevention and management of pressure injuries. Their dual role—as healthcare professionals and trusted members of the communities they serve—positions them uniquely to deliver timely, context-sensitive interventions.

However, findings from this study revealed that the professional expertise and responsiveness of community nurses in addressing pressure injuries were not prominently reflected. Moreover, their role in fostering strong connections with residents was not effectively demonstrated. This indicates a gap in current practice and suggests a need to strengthen the involvement of community nurses in both the clinical and relational aspects of pressure injury care.

To address this gap, future strategies should focus on empowering community nurses with adequate resources, continuous training, and institutional support to enable their proactive engagement in pressure injury prevention and management. In particular, their roles should be expanded to include: (1) providing ongoing educational support to family caregivers through home visits or teleconsultation; (2) performing risk assessments and early identification of pressure injuries in home-based settings; (3) serving as liaisons between hospitals and community resources to ensure continuity of care; and (4) offering emotional support and counseling to alleviate caregiver burden. These expanded responsibilities are essential for enhancing the effectiveness of home-based care and reducing preventable complications.

Integrating these efforts with technology-based health education platforms may further improve outreach, promote care continuity, and ensure that interventions are tailored to the specific needs of each community.

## Limitations

This study has several limitations. Firstly, during data collection, we intended to conduct as many family observations as possible. However, these observation sites were limited to a community in Suzhou due to financial constraints. Future studies should be conducted in a variety of geographical settings. Secondly, this is a qualitative study, thus quantitative conclusions cannot be made. Thirdly, the involvement of multiple researchers in conducting the interviews and observations can present a limitation, as it may introduce variability in data collection. However, to minimize this potential bias, we implemented several measures: all researchers received standardized training prior to data collection to ensure consistency, a subset of data was independently coded by multiple researchers to check for agreement, and regular team discussions were held to address any discrepancies. Furthermore, while the translated guideline (Chinese version) ensures a certain degree of cultural adaptation, further attention to transcultural aspects is important when evaluating caregiver behaviors in different social and cultural contexts.

## Conclusion

Eight themes were extracted after analysing the on-site observation records and interviews. These included four topics on care content (food preparation and feeding support, maintaining hygiene, postural change and use of support, use of medication, and dressing), two perception topics (responsible care and compensatory companion, grief, and helplessness), and two challenge topics (identifying and responding to early signs of pressure injury and shortage of professional care and counselling resources). Family caregivers still engage in inappropriate care behaviours, such as in terms of the frequency of body position changes and ways to keep the wound clean and hygienic. The education of family caregivers of disabled older adults should include the identification of pressure injuries and related early warning signs and introduce cases for teaching purposes when appropriate. To promote the availability of professional care and counselling resources for pressure injuries, the community can be united to carry out systematic science popularisation by using various effective media such as new media and video channels.

## Electronic supplementary material

Below is the link to the electronic supplementary material.

Supplementary Material 1

Supplementary Material 2

## Data Availability

The datasets generated and analyzed during the current study are not publicly available due to confidentiality requirements but are available from the corresponding author upon reasonable request.

## Declarations

### Authors’ information

Yi Chen is a member of the World Council Enterostomal Therapists (WCET) and serves as the secretary of the Society of Wound, Ostomy and Incontinence Nursing in Suzhou. She worked at Soochow University during the year 2022–2024, and is now studying for her doctorate at Fudan University.

## Abbreviations

CAPI: community-acquired pressure injuries
WCET: World Council Enterostomal Therapists
