# Supplementary material for: Family caregivers’ perceptions and challenges in the care of pressure injuries in daily life: a qualitative study
Source: BMC Geriatr. 2025 Jul 3;25:490. doi: 10.1186/s12877-025-06114-1 (PMC12232193; doi:10.1186/s12877-025-06114-1)
Supplement: Supplementary file 1 — Supplementary Material 1 [file 12877_2025_6114_MOESM1_ESM.docx]

**Interview Guide for Family Caregivers**

**1.Can you tell me about the main work that you do every day to care for patients with this pressure injury?**

**2. Please talk about your daily life when you were not caring for the patient with pressure injury. For example, food, clothing, social activities, transportation?**

**3. How do you identify pressure injuries? What changes do you think may alert you that the patient might have suffered from pressure injury?**

**4. What are the main changes in your daily life as a caregiver after the patient suffering from the pressure injury?**

**5. Could you please talk something about how caring for patients has affected you mentally, physically and emotionally? Furthermore, can you tell me specifically what kind of burden has been added to you?**

**6. Did you experience any changes in mood before and after the patient’ s pressure injury appeared?**

**7. What is your current physical condition? Is there any change before and after the patient’ s pressure injury appeared?**
