# Supplementary material for: Family caregivers’ perceptions and challenges in the care of pressure injuries in daily life: a qualitative study
Source: BMC Geriatr. 2025 Jul 3;25:490. doi: 10.1186/s12877-025-06114-1 (PMC12232193; doi:10.1186/s12877-025-06114-1)
Supplement: Supplementary file 2 — Supplementary Material 2 [file 12877_2025_6114_MOESM2_ESM.docx]

**Supplementary File 2. Observation Script for the qualitative study of Family Caregivers in Pressure Injury Care**

This observation script was developed to guide participant observation of family caregivers providing care for adults with pressure injuries in community settings. It served as a semi-structured, flexible framework to ensure systematic yet responsive data collection during fieldwork.

**I. Observation Stages**

**1. Descriptive Observation**

• Conducted during the initial entry into the field.

• Aimed at building rapport with patients and family caregivers.

• Documenting the overall caregiving environment and routine workflows.

**2.** **Focused Observation**

• Conducted following initial interaction with caregivers.

• Each caregiver was observed once for 2–3 hours, aligned with caregivers’ daily routines (typically between 07:30 and 18:30).

• Targeted morning or evening care activities, including feeding, repositioning, and hygiene support.

**3.** **Selective Observation**

• Focused specifically on pressure injury-related caregiving behaviors.

• Grounded in the *2019 Chinese version of the Prevention and Treatment of Pressure Ulcers/Injuries Guidelines* (adapted from the European Pressure Ulcer Advisory Panel).

**II. Core Observation Domains**

- **Pressure Injury Prevention and Management**

• Use of pressure-relieving devices (e.g., cushions, mattresses). ____________________

• Repositioning frequency and techniques. _________________________________________

• Dressing application and wound care routines.

• Skin inspection and protection of vulnerable areas.

Key points_______________________________________________________

- **Nutritional Support**

• Meal preparation, timing, and delivery.

• Feeding assistance strategies. _____________________________________________________

• Observation of patients’ appetite and intake. _______________________________

• Use of nutrition supplements, if applicable. _______________________________

Key points_______________________________________________________

- **Interaction with Healthcare Professionals**

• Involvement of community nurses, physicians, or wound care specialists. _______

• Implementation of professional care advice. _______________________________

• Communication patterns between caregivers and providers. ____________________

Key points_______________________________________________________

- **Caregiver–Patient Interactions**

• Verbal and non-verbal communication.

• Emotional tone (e.g., patience, frustration, empathy).

• Caregiver’s response to patient discomfort or non-compliance.

Key points_______________________________________________________

- **Daily Caregiving Workflow**

• Scheduling and prioritization of tasks.

• Use of assistive tools or devices (e.g., gloves, bedpans).

• Time allocation for different care tasks.

• Coexistence of caregiving with other household responsibilities.

Key points_______________________________________________________

- **Environmental and Contextual Factors**

• Physical space (e.g., cleanliness, mobility accessibility).

• Availability of supplies and equipment.

• Presence of other family members or support networks.

• Socioeconomic or cultural factors influencing care.

Key points_______________________________________________________

**III. Data Recording Procedures**

• Observers used shorthand field notes to record observations in real time.

• Audio recordings and photographs were taken with prior informed consent.

• After each session, observers prepared reflective memos summarizing impressions, emotional tone, and contextual insights.

• This standardized script was used to guide the observation while allowing flexibility to capture emergent themes.

**IV. Ethical Considerations**

• Observers maintained a non-intrusive presence and did not intervene in caregiving activities.

• Clarifying questions were asked sparingly to understand decision-making, without disrupting routines.

• Participants were informed of the observation but not the specific research objectives, to ensure authenticity in behavior.

• All observers were trained and followed confidentiality and ethical protocols.
